# Hilar cholangiocarcinoma: Controversies on the extent of surgical resection aiming at cure

**DOI:** 10.1007/s00384-014-2063-z

**Published:** 2014-11-08

**Authors:** Shuai Xiang, Wan Yee Lau, Xiao-ping Chen

**Affiliations:** 1 Hepatic Surgery Centre, Tongji Hospital, Tongji Medical College, Huazhong University of Science and Technology, Wuhan, Hubei China; 2 Faculty of Medicine, The Chinese University of Hong Kong, Shatin, New Territories, Hong Kong, SAR China

**Keywords:** Hilar cholangiocarcinoma, Negative margin, Hepatic resection, Vascular resection

## Abstract

**Background:**

Hilar cholangiocarcinoma is the most common malignant tumor affecting the extrahepatic bile duct. Surgical treatment offers the only possibility of cure, and it requires removal of all tumoral tissues with adequate resection margins. The aims of this review are to summarize the findings and to discuss the controversies on the extent of surgical resection aiming at cure for hilar cholangiocarcinoma.

**Methods:**

The English medical literatures on hilar cholangiocarcinoma were studied to review on the relevance of adequate resection margins, routine caudate lobe resection, extent of liver resection, and combined vascular resection on perioperative and long-term survival outcomes of patients with resectable hilar cholangiocarcinoma.

**Results:**

Complete resection of tumor represents the most important prognostic factor of long-term survival for hilar cholangiocarcinoma. The primary aim of surgery is to achieve R0 resection. When R1 resection is shown intraoperatively, further resection is recommended. Combined hepatic resection is now generally accepted as a standard procedure even for Bismuth type I/II tumors. Routine caudate lobe resection is also advocated for cure. The extent of hepatic resection remains controversial. Most surgeons recommend major hepatic resection. However, minor hepatic resection has also been advocated in most patients. The decision to carry out right- or left-sided hepatectomy is made according to the predominant site of the lesion. Portal vein resection should be considered when its involvement by tumor is suspected.

**Conclusion:**

The curative treatment of hilar cholangiocarcinoma remains challenging. Advances in hepatobiliary techniques have improved the perioperative and long-term survival outcomes of this tumor.

## Introduction

Hilar cholangiocarcinoma, or Klatskin tumor, is the most common malignant tumor affecting the extrahepatic bile duct. It is relatively slow growing and is usually small at clinical presentation. Only a very few patients with unresectable, indolent, and slow-growing hilar cholangiocarcinoma can have long-term survival [1]. The prognosis of most patients with unresectable tumor is poor because of the vital position of the tumor, with a median survival of less than 1 year. The treatment for hilar cholangiocarcinoma is challenging. Surgical resection remains the only potentially curative therapy. The survival rates for patients who had received R1 or R2 resection were significantly better than those with unresectable tumors [2–4]. In the old days, surgical treatment of hilar cholangiocarcinoma aimed mainly at obtaining a diagnosis through laparotomy and relieving obstructive jaundice through surgical intubation or internal bypass [5]. In the past three decades, the extent of surgical resection for hilar cholangiocarcinoma has shifted from local excision of the affected bile duct at the liver hilum with a local cone of adjacent liver parenchyma to more extensive resections involving combined major liver resection with increased long-term survival rates and decreased mortality and morbidity [6–10]. It is now widely accepted that resection of the bile duct cannot be accepted as a curative operation, and radical resection with combined hepatectomy is now adopted by most surgeons. The key strategy of surgical resection is to achieve adequate resection margins. This article focuses on the controversies in radical surgical resection of hilar cholangiocarcinoma, including issues about the relevance of adequate resection margins, routine caudate lobe resection, extent of liver resection, and combined vascular resection on perioperative and long-term survival outcomes of patients with resectable hilar cholangiocarcinoma.

## Materials and methods

English medical literatures were extensively searched via online pubmed using the search strategy “hilar cholangiocarcinoma OR Klatskin tumor”. Studies focusing on curative surgical resection for hilar cholangiocarcinoma were included. Case report and studies with less than 40 resections were excluded. Outcomes of surgical resection from 73 studies published during the period 1990–2014 were extracted and summarized in Table 1. Correlation was analyzed by Pearson’s test. A *P* value of <0.05 was considered statistically significant.

**Table 1:** Outcomes of resection for hilar cholangiocarcinoma in series with more than 40 resections

Authors	Published Year	Resections	R0 (%)	PH (%)	Mortality (%)	Morbidity (%)	5-year survival (%)	R0 5-year survival (%)	PVR (%)	AR (%)	CR (%)	Resectablity* (%)	Bismuth III and IV (%)
Nimura et al.	1990	55	84	93	11	35	38	41	25	NA	82	83	NA
Gazzaniga et al.	1993	48	NA	NA	23	79	NA	20	NA	NA	NA	NA	NA
Ogura et al.	1993	55	51	60	2	22	23	NA	9	2	51	96	69
Sugiura et al.	1993	83	57	100	8	NA	20	33	22	5	67	NA	NA
Su et al.	1996	49	49	57	10	47	15	NA	2	NA	22	NA	61
Nakeeb et al.	1996	109	26	14	4	39	11	NA	NA	NA	NA	NA	NA
Klempnauer et al.	1997	151	77	77	10	NA	28	NA	26	1	27	NA	82
Miyazaki et al.	1998	76	71	86	13	33	26	40	26	9	86	NA	NA
Launois et al.	1999	40	80	75	13	25	13	NA	18	NA	23	43	78
Kosuge et al.	1999	65	52	88	9	37	33	NA	5	5	83	73	NA
Neuhaus et al.	1999	80	55	83	8	NA	22	37	29	NA	83	NA	83
Miyazaki et al.	1999	93	70	86	10	38	26	NA	26	9	86	NA	NA
Todoroki et al.	2000	101	14	58	4	14	28	67	NA	NA	NA	89	70
Lee et al.	2000	111	78	100	6	48	22	NA	26	4	100	NA	NA
Gerhards et al.	2000	112	14	29	18	65	NA	NA	9	8	NA	NA	53
Nimura et al.	2000	142	76	90	9	49	NA	26	30	NA	NA	NA	NA
Jarnagin et al.	2001	80	78	78	10	64	27	30	11	NA	28	50	NA
Kawarada et al.	2002	87	64	75	2	28	26	NA	NA	NA	69	89	NA
Shimada et al.	2003	53	66	77	9	45	15–25	34	19	13	74	87	49
Seyama et al.	2003	58	64	100	0	43	40	46	16	NA	100	94	71
Kawasaki et al.	2003	79	68	96	1	14	22	40	6	3	87	75	78
Kondo et al.	2004	40	95	78	0	48	NA	NA	25	25	78	93	53
Jitsma et al.	2004	42	65	100	12	45	22	45	17	10	57	NA	NA
Rea et al.	2004	46	80	100	9	52	26	30	NA	NA	39	NA	85
Ramesh et al.	2004	46	70	76	7	28	22	25	7	NA	76	36	52
Hemming et al.	2005	53	80	98	9	40	35	45	43	6	98	66	NA
Jarnagin et al.	2005	106	77	82	8	62	NA	NA	9	NA	34	49	NA
Dinant et al.	2006	99	31	38	15	66	27	33	7	NA	15	NA	55
Sano et al.	2006	102	61	100	0	50	44	NA	22	5	100	92	NA
Hasegawa et al.	2007	49	78	90	2	47	40	NA	6	0	90	NA	84
Baton et al.	2007	59	68	100	5	42	20	28	8	2	100	NA	NA
Hidalgo et al.	2008	44	45	93	7	59	28	45	45	9	77	90	NA
Konstadoulakis et al.	2008	59	66	86	7	25	34	NA	24	NA	64	81	86
Endo et al.	2008	101	81	82	5	NA	31	NA	9	NA	36	NA	NA
Murakami et al.	2009	42	74	86	7	52	30	NA	NA	NA	90	NA	NA
Young et al.	2009	51	57	92	8	75	20	40	41	10	92	NA	92
Miyazaki et al.	2009	107	59	91	2	NA	28	33	23	3	NA	NA	83
Chen et al.	2009	138	89	100	0	30	27–34	NA	33	NA	92	74	67
Hirano et al.	2009	146	87	88	3	44	36	NA	45	14	88	NA	62
Giuliante et al.	2010	43	77	93	7	53	36	NA	NA	1	NA	29	NA
Ercolani et al.	2010	51	73	100	10	51	34	44	8	4	78	82	100
Rocha et al.	2010	60	80	78	5	35	NA	54	NA	NA	48	57	NA
Gulik et al.	2010	99	31	38	10^§^	68^§^	NA	NA	18	NA	39	NA	NA
Kobayashi et al.	2010	119	66	92	NA	NA	NA	NA	NA	NA	NA	NA	NA
Unno et al.	2010	125	63	95	8	49	35	45	34	3	100	NA	80
Igami et al.	2010	298	74	98	2	43	42	52	37	18	98	NA	88
Lee et al.	2010	302	71	89	2	NA	33	47	13	2	89	86	81
Gulik et al.	2011	41	92	85	7	NA	NA	NA	22	NA	78	NA	78
Saxena et al.	2011	42	64	100	2	45	24	NA	26	5	36	78	79
Chauhan et al.	2011	51	73	76	12	69	29	NA	6	NA	8	NA	NA
Guglielmi et al.	2011	62	74	87	10	55	15	NA	16	3	76	NA	NA
Hemming et al.	2011	95	84	100	5	34	43	50	44	5	100	NA	89
Neuhaus et al.	2011	100	NA	100	11–12	NA	43	NA	NA	NA	NA	NA	90
Otto et al.	2011	123	79	89	6	NA	26	NA	38	NA	89	77	91
Li et al.	2011	215	66	95	5	NA	30	41	16	6	NA	73	41
Ruys et al.	2012	57	75	88	NA	NA	42	NA	NA	NA	NA	60	NA
Cannon et al.	2012	59	63	83	5	39	NA	58	NA	NA	NA	54	NA
Cho et al.	2012	105	71	78	14	NA	34	50	8	NA	59	NA	81
Kow et al.	2012	127	89	97	2	6	30–66	NA	NA	NA	55	NA	100
Matsuo et al.	2012	157	76	82	8	59	32	36	10	NA	36	53	NA
Lee et al.	2012	162	77	81	1	NA	42	45	6	2	76	66	69
Cheng et al.	2012	171	78	100	3	26	14	17	13	3	80	61	100
de Jong et al.	2012	305	65	73	12	NA	20	25	17	NA	NA	NA	44
Nuzzo et al.	2012	440	77	85	9	37	26	32	8	2	67	NA	59
Nagino et al.	2012	574	77	97	5	57	33	NA	36	13	97	90	85
Song et al.	2013	230	77	78	4	NA	33	40	10	1	44	100	70
Dumitrascu et al.	2013	90	76	73	8	53	27	31	13	3	50	69	76
Farges et al.	2013	366	NA	100	11	69	NA	NA	23	NA	NA	NA	78
Gomez	2014	57	74	86	14	60	40	NA	9	4	86	67	NA
Yu	2014	238	50	51	1	18	17	NA	11^¶^	20	NA	NA	86
Furusawa et al.	2014	144	74	99	1	73	NA	NA	15	3	100	NA	78
Tamoto	2014	49	82	100	4	63	NA	NA	73	NA	100	NA	NA

^*^Data indicates percentage of resections in patients explored with curative intent

^§^In the last period (1998–2003, *n* = 29)

^¶^Data indicates percentage of portal vein resections in R0 resections

PVR indicates portal vein resecion

*AR* artery resecion; *CR* caudate lobe resecion; *NA* data not available

## Results and discussion

### Tumor-free resection

The most important factor affecting long-term survival in the surgical treatment of hilar cholangiocarcinoma is whether the tumor has been completely resected on histological examination (R0 resection). The margin status include R0 margin (no residual tumor), R1 margin (microscopic residual tumor), and R2 margin (macroscopic residual tumor). Patients with R1 margin or R2 margin have a dismal survival [11, 12]. Of the many clinicopathological factors affecting long-term survivals, R0 resection is the only factor which can be modified by the surgeon. Thus, the primary goal of surgical therapy is to achieve R0 resection [13].

Several reports have suggested that the long-term survivals after R0 and R1 were not significantly different [14–16]. There is a possibility that in these reports, some of the patients with R1 resection were mistakenly classified as R0 resection because there was no adequate sampling of the margins, especially in patients with narrow resection margins. Endo et al. classified their patients who have received R0 resection into the wide margin group and the narrow margin group. Of all the patients with R0 bile duct margins shown intraoperatively, only 60 % were associated with improvement in disease-specific survival when compared with patients with R1 resections. While the group of patients with wide margin experienced better, the group of patients with narrow margin had similar disease-specific survival similar to those patients who underwent R1 resection [17]. Similarly, Seyama et al. found that patients with surgical tumor-free margin of over 5 mm resulted in significantly better long-term survival than those patients with a margin of less than 5 mm. However, there was no difference between the survival of patients after R0 resection with those who had a narrow margin (<5 mm) or those who had received R1 resection [15]. The wider and the longer the resection margin, the less likely it is to find a positive resection margin [18]. Thus, wide and long R0 margins are required for resection with curative intent in hilar cholangiocarcinoma resectional surgery.

It is frequently difficult to achieve a wide and long resectional margin for curative treatment. First, hilar cholangiocarcinoma is located in the liver hilum surrounded by vital structures. Second, it is difficult to determine the exact length and width of microscopic tumor extension preoperatively and intraoperatively. The biological nature of cholangiocarcinoma involves proximal microscopic spread of the disease along the bile duct extending beyond the palpable macroscopic boundaries of the primary hilar mass. Sakamoto et al. histologically examined serial sections of 62 specimens of resected hilar cholangiocarcinoma. They found that anastomotic recurrences never occurred in patients who had a proximal tumor-free resection margin greater than 5 mm, suggesting that a 5 mm tumor-free margin was adequate for curative intent [19]. However, it has been demonstrated that the longitudinal extent of tumor at the proximal border ranged from 0.6 to 18.8 mm in the submucosa layer [19], and that the width of the superficial extension showed a wide distribution of 31–52 mm [18, 19]. Surgeons, thus, cannot be certain on the length and width of the resection margins. Third, intraoperative frozen-section examination of ductal margins has an accuracy, sensitivity, and specificity of only 56.5, 75.0, and 46.7 %, respectively [20].

Nonetheless, to achieve real R0 resection, transection of the proximal bile duct above the macroscopic border of the primary tumor should be carried out as high as technically feasible with careful consideration of the potential morbidity, and resection of hilar cholangiocarcinoma combined with major hepatectomy has the potential to provide wide and long resection margins on the ipsilateral side of the combined liver resection [17].

### Further resection

The recent reported incidences of positive resection margins in patients who had undergone surgical resection with curative intent ranged from 64.6 to 88.2 % at high-volume centers [4, 10, 14, 21–23]. When a positive resection margin is diagnosed intraoperatively using frozen section examination, further resection is recommended if technically possible to obtain complete tumor removal [11, 12, 24]. However, further resection of the bile duct at the proximal side can be technically difficult due to encroachment onto vital structures and adjacent liver parenchyma [2, 25, 26]. Not every patient with R1 resection can be subjected to further resection [17, 25]. For patient who can be further resected because of technically feasibility or no radial tumoral invasion, 54 to 83 % can achieve R0 resection [12, 14, 17, 25]. Unfortunately, the clinical usefulness of further resection has not yet been established, and it is still controversial whether further resection improves patients’ survival [12, 14, 17, 25]. While several reports have suggested that further resection did not contribute to improvement in survival [14, 17] probably because of the limited further length of less than 5 mm that could be resected [25], Ribero and colleagues achieved good results in 15 secondary negative margins in 18 additional resections [12]. Unfortunately, they did not record the exact length of further bile duct resection. However, they demonstrated that the survival of patients who had a secondary R0 resection was similar to that of the primarily R0-resected patients, and they were significantly better than those patients with R1 resection. They also showed similar median survival to recurrence and similar incidence of local site recurrence when patients with R1 resection were compared with patients with R0 resection, independent of whether further resection was carried out [12]. Notably, the primary essence of further resection is to obtain tumor-free margins wide and long enough for curative intent. It is recommended that more extended resections with adequate surgical margins should be carried out when technically possible and when the patient has good functional reserves [25]. To further address these issues, randomized controlled studies with adequate patients are required, and the adequate length of bile duct resection should be carefully defined to provide more convincing data.

### Hepatic resection

In the old days, hilar cholangiocarcinoma had been considered to be unresectable and palliative decompression of the obstructed biliary tract using bypass surgery or tube-drainage was popularly employed. Surgical resections with curative intent were later attempted to achieve better survival [27–31]. Both local resection of bile duct and resection combined with major hepatectomy were used. The surgical strategy was made according to the location and involvement of the tumor mass. When the tumor was small or localized, the tumor was resected together with an adjacent cone of liver parenchyma. If it infiltrated into the liver parenchyma, combined major liver resection was carried out [24, 27, 32, 33].

In 1990, a review on 581 resections for hilar bile duct cancer was published: 245 patients (42.2 %) received local bile duct confluence resection while 224 (38.6 %) patients received resection of the bile duct confluence combined with major liver resection [34]. Tumor cells from hilar cholangiocarcinoma are apt to infiltrate along the bile duct wall and invade into the surrounding vital structures as well as the adjacent liver parenchyma because the confluence of the bile ducts has thin walls and it is strategically situated. Even as early as the 1980s [7, 31, 34], surgeons started to hold the view that hilar cholangiocarcinoma should be regarded more as a regional than a local disease. As a consequence, an increasing number of combined major liver resections were performed [34]. However, there was no survival benefit at that time using bile duct resection combined with major hepatectomy when compared with local resection of a cone of adjacent liver parenchyma [33, 35]. The increase in 5-year survival rate from 6 % for local resection to 14 % for major liver resections was almost completely offset by the increase in postoperative mortality from 9 % for local resections to 17 % for major liver resections [34]. Also, the mean survival time [34] as well as the R0 resection rate [33, 35] had hardly been improved by the use of combined major liver resection.

**Fig. 1:**
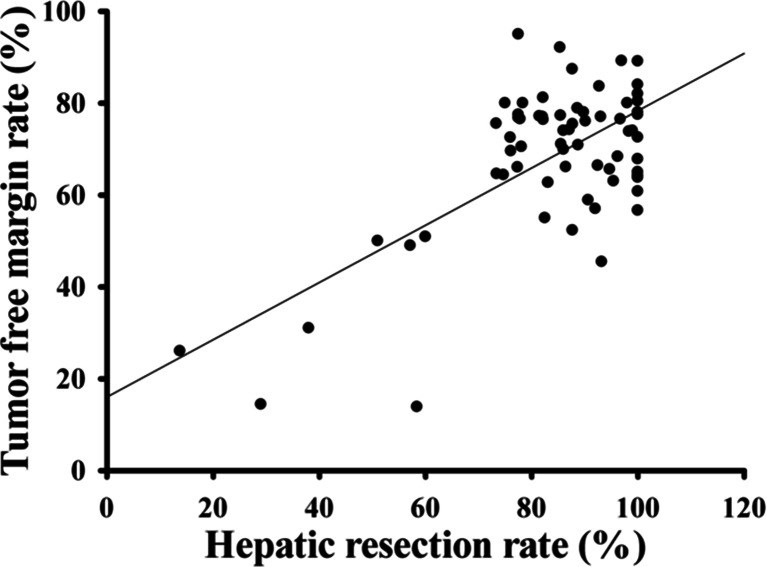
Positive correlation of hepatic resection rates and tumor-free margin achievement. Data were extracted from Table 1

Over the last two decades, complex and aggressive surgical resections were made possible with acceptable morbidity and mortality rates by major advances in patient selection, radiologic assessment, surgical techniques, and perioperative care [21, 33, 36–41]. At the same time, there are more evidences to support bile duct resection with an adjacent cone of liver parenchyma cannot be accepted as an adequate curative treatment, and combined major hepatic resection is associated with improved survival [38, 39, 42–44]. The main aim of the aggressive surgical approach is to obtain R0 resection. Figure 1 shows that the percentage of hilar cholangiocarcinoma resected with combined major hepatic resection in surgical series is positively correlated with the tumor-free resection margin rate. Most reports coming from single center studies indicated that there was a progressive increase in proportion of patients submitted to combined major hepatic resection with an increased R0 resection rate and improved survival over the study period [3, 37, 45–50] (Table 2). Bile duct resection combined with major hepatic resection has now been increasingly accepted as a standard surgical treatment for hilar cholangiocarcinoma [51].

**Table 2:** Surgery outcomes according to the time period in several series from single centers

Authors	Published year	Time period		Resections	R0 (%)	PH (%)	Mortality (%)	Morbidity (%)	5-year survival (%)
Gerhards et al.	2000	1983	−1987	42	5	36	19	66	NA
		1988	−1992	45	13	9	20	73	NA
		1993	−1997	25	32	52	12	52	NA
Kawarada et al.	2002	1976	−1993	62	55	65	2	31	20
		1994	−2000	25	88	100	4	20	50
Dinant et al.	2006	1988	−1993	45	13	9	20	73	22
		1993	−1998	25	32	52	12	52	35
		1998	−2003	29	59	72	10	68	59
Gulik et al.	2010	1988	−1993	45	13	9	NA	NA	20
		1993	−1998	25	32	52	NA	NA	
		1998	−2003	29	59	72	10	68	33
Nagino et al.	2012	1977	−1990	72	75	92	11	76	23
		1991	−2000	116		93	10	80	
		2001	−2005	168	78	98	3	52	38
		2006	−2010	218		99	1	43	
Furusawa et al.	2014	1990	−2000	70	70	99	1.4	85.7	33
		2001	−2012	74	78	100	0	61	35

NA indicates data not available

#### Is hepatic resection necessary for type I or II hilar cholangiocarcinoma

Even though combined major hepatic resection is widely accepted for Bismuth type III and IV hilar cholangiocarcinoma, whether it is necessary for type I or II tumor is still controversial. A number of researchers consider that tumor resection with an adjacent cone of liver parenchyma is sufficient for patients with type I or II tumor [10, 52–55] especially for type I tumors [10, 21, 56, 57]. Launois et al. performed combined major hepatectomy depending on two factors: tumor location and TNM classification [58]. They performed local hilar resection mainly for Bismuth I or II tumors with Tis and T1 lesions and the survival seemed better than that of combined major hepatectomy which was done mainly for Bismuth III or IV tumors, suggesting that local hilar resection is sufficient if the tumor is Bismuth I or II [58]. Similarly, Otani et al. compared local hilar resection for Bismuth type I or II tumors with T2 or less lesions with combined major hepatectomy for type III or IV tumors and found that similar R0 resection rates as well as cumulative survivals were equally achieved in the two groups [59]. They concluded that local hilar resection was indicated for papillary T1 or T2 tumors in Bismuth type I or II tumor [59]. However, there are limited data to assess the effectiveness of combined major hepatic resection for type I or II tumor in these studies, and it is difficult for a surgeon to get the precise information on the tumor classification or the TNM staging preoperatively or intraoperatively [60]. Ikeyama et al. compared survival in patients with nodular and infiltrating hilar cholangiocarcinoma who tolerated right hemihepatectomy with survival in patients who tolerated bile duct resection with or without limited hepatic resection, and recommended that the surgical approach to Bismuth type I and II hilar cholangiocarcinoma should be determined according to preoperative cholangiographic features. For nodular and infiltrating hilar cholangiocarcinoma, right hepatectomy is essential for cure, for papillary tumor local resection with or without limited hepatic resection is adequate [48]. A more recent retrospective study, which aimed to evaluate surgical outcomes of bile duct resection alone and combined major liver resection in 52 patients with Bismuth type I and II hilar cholangiocarcinoma, revealed that concomitant liver resection had a higher curability, lower local recurrence rate, and better overall survival with a similar postoperative morbidity and mortality [61]. In addition, the authors found that cancer recurred in three patients out of the six R0 resectional papillary tumors treated by bile duct resection alone [61]. It seems that concomitant liver resection should be considered in all patients with Bismuth type I and II hilar cholangiocarcinoma regardless of the tumor classification. Several types of hepatic resections were performed in that study, including left or right hepatectomy or volume-preserving liver resection [61]. In our experience, central hepatectomy resecting segment 5 and segment 4b/extended 4b resection is the primary choice for type I and II tumors [62]. This operation is adequate for both negative resection margins and good exposure. Properly conducted prospective randomized controlled trials are needed to validate the treatment strategy for type I and II hilar cholangiocarcinoma.

#### Routine caudate lobe resection

The caudate lobe is located deep in the liver between the inferior vena cava and the hepatic hilum, thus isolation and resection of the caudate lobe remain a challenge for surgeons. The importance of tumor involvement of the caudate lobe in the treatment of hilar cholangiocarcinoma had not been fully recognized two decades ago. Some surgeons do not adopt a strategy of routine caudate lobe resection even now [33, 35], mainly due to the deep anatomical location of the caudate lobe and the concern on postoperative insufficient remnant liver parenchyma. The result was dismal. Bengmark et al. employed major liver resections in 100 % of the 22 patients with hilar cholangiocarcinoma from 1968 to 1984; however, they did not emphasize the inclusion of caudate lobe resection in the surgical procedure [7]. R0 resection was achieved just in 18.2 % of the patients [7].

**Fig. 2:**
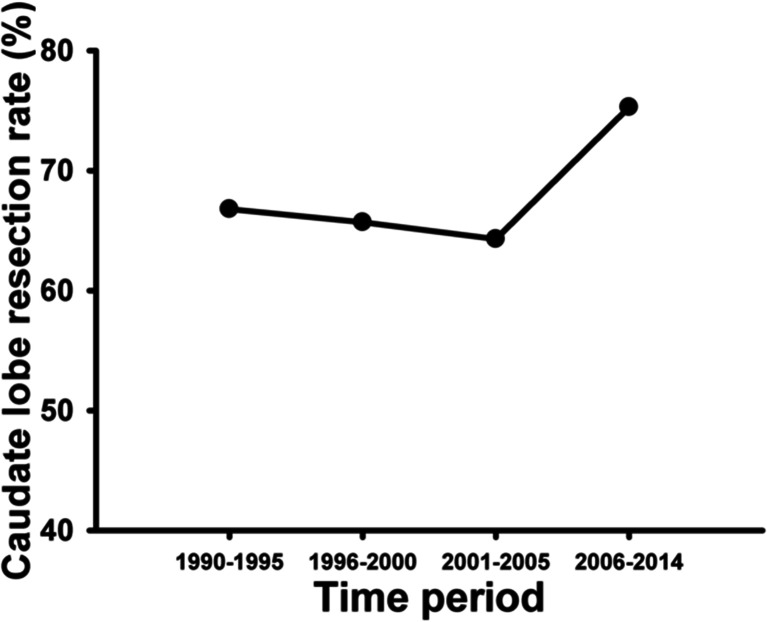
Caudate lobe resection rate according to the time period. Data were extracted from Table 1

The close anatomic relationship between the caudate lobe and the hilar cholangiocarcinoma was well studied by Mizumoto et al. in 1986 [63]. As hilar cholangiocarcinoma has a high chance to invade the biliary branches or directly infiltrate the parenchyma of the caudate lobe [38–40, 64], routine caudate lobe resection should be carried out for curative treatment of hilar cholangiocarcinoma [63]. Better R0 resection rates and higher cumulative survival rates are achieved by concomitant caudate lobe resection [9, 39, 49]. At present, caudate lobe resection is increasing carried out for hilar cholangiocarcinoma all over the world [9, 45, 49, 65]. Table 1 shows that, for the period of 2006 to 2014, there were 3447 caudate lobe resections in 4577 patients with surgical resection for hilar cholangiocarcinoma, which are significantly higher than the percentage of caudate lobe resection carried out from 1980 to 2005 (Fig. 2). Two retrospective studies published in 2012 aimed primarily to assess the specific role of routine caudate lobe resection for Bismuth type III/IV hilar cholangiocarcinoma found total caudate lobectomy contributed to improvement in survival [22, 66]. The role of routine caudate lobe resection in Bismuth type I/II tumors is still uncertain.

Nimura et al. reported in 1990 that 98 % of caudate lobe resections were histologically confirmed to be tumor positive [64]. However, other authors reported that the caudate lobe was involved in hilar cholangiocarcinoma in 32.4 ± 7.1 % (mean ± standard deviation) [38, 39, 45, 62, 67, 68]. It is our opinion that it should be routine as at least one third of patients had the caudate lobe involved by hilar cholangiocarcinoma.

#### Major liver resection or minor liver resection

There are still controversies on the optimal extent of hepatic resection to achieve a high percentage of R0 resection for hilar cholangiocarcinoma.

Combined major liver resection represents an aggressive surgical approach to remove a large volume of hepatic parenchyma, including the use of right trisectionectomy (Couinaud segment 1, 4–8), right hemihepatectomy (S1, 5–8), left trisectionectomy (S1–5, S8), or left hemihepatectomy (S1–4). This approach has widely been advocated as a prime choice of surgical treatment for hilar cholangiocarcinoma, especially in patients with advanced tumors [69–71]. It is believed that combined major liver resection has the advantage to increase surgical curability by obtaining wide and negative surgical resection margins [72, 73]. In addition, hemihepatectomy/trisectionectomy is technically feasible and can be carried out by many surgeons. The major drawback of combined major liver resection is the small postoperative liver remnant which is associated with high surgical morbidities and mortalities [44, 49, 74, 75]. Dinant et al. reported hospital morbidity of 70.3 % (26/37) and mortality of 21.6 % (8 dead in 37) in patients after hemi- or extended hemihepatectomy [49]. Similarly, Ramesh et al. and Gerhards et al. reported that the overall mortality rate was 25 % (3/12 and 8/32, respectively) after major liver resections resulting in postoperative liver failure [50, 75]. Thus, the increase in resectability rate can be offset by the increase in postoperative mortality after associated major liver resection. Some approaches have been proposed to reduce the perioperative risk of associated major liver resection, including adequate assessment of the volume/function of the future remnant liver, preoperative biliary drainage, and portal vein embolization (PVE). Kawasaki et al. showed an in-hospital mortality rate as low as 1.3 % could be achieved after extended, mainly right, hepatectomy with routine preoperative biliary drainage and hemihepatic PVE [60]. Preoperative PVE is thought to be effective to induce hypertrophy in the remnant liver and thus may increase safety for patient who is considered insufficient in remnant liver volume. However, the benefit of preoperative biliary drainage and PVE has not been fully recognized and consensus of indication criteria has not yet been established [52, 60, 71, 76–78]. PVE procedure may delay the surgical resection and associate with rapid tumor growth or liver metastases. Besides, the estimated blood loss and operation time were reported significantly higher in PVE group [52].

**Fig. 3:**
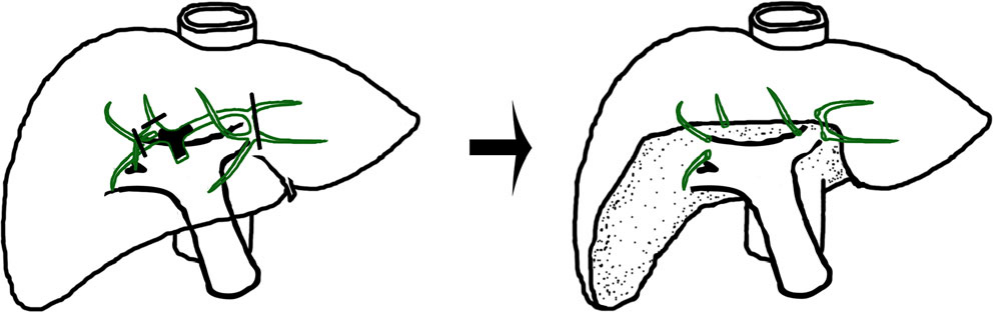
Minor liver resection. Liver parenchyma adjacent to the liver hilum, including segment 1, 4b, 5, and part of segment 6, 7, 8, is resected. Resection of segment 4b and 5 provides good exposure

In the late twentieth century, Nimura and Miyazaki advocated using minor central hepatic resection in carefully selected patients to preserve as much as possible the functional liver volume [44, 64]. Limited central liver resection means excision of liver segments/subsegments around the liver hilum, such as segment 1 resection, segment 1 and 4 (4b) resection, segment 1, 4 (4b), and 5 resection, or mesohepatectomy (segment 1, 4 (4b), 5, and 8 resection). We believe that this approach should take an important part in surgical treatment for most of the hilar cholangiocarcinoma. First, a large amount of liver parenchyma involved in combined major liver resection is free of tumor and it is not necessarily to resect these liver tissues. Based on a three-dimensional perception of the tumor located centrally in the liver, the aim of the curative resection is to resect adequately the bile duct bifurcation with the adjacent liver parenchyma. Generally, resection of segment 1, 4 (4b), and 5 is adequate. The extent of liver resection can occasionally be modified to include partial segment 6, 7, and 8 if necessary (Fig. 3). Minor central liver resection can be performed in patients with type I, type II, type IIIa, and type IIIb tumors [62] and even for type IV tumors [56]. Second, although there are some concerns that minor central liver resection may decrease the rate of curative surgical resection, the Japanese surgeons have shown clearly that surgical curability and postoperative survival rates in selected patients with minor central hepatic resection were not compromised and surgical morbidity/mortality rate was significantly lower than that in the combined major liver resection group [44, 79, 80]. Similarly, patients who received minor central liver resection had good perioperative outcomes (9.7 % morbidity and 0 % mortality) with no decreased long-term survival rates (5-year survival rates were 34 % for minor resection vs. 27 % for major resection) [62]. One should be noticed that there is an intrinsic limitation in these retrospective studies. Patients submitted in major or minor resection groups were not randomized controlled. Select bias occurred when the surgeons made the decision of surgical strategy. Minor resections tend to be chosen for type I to III tumors or tumors confined to the first-order hepatic duct. Thus, it is difficult to tell the differences of these two surgical strategies in a tumor with a certain bismuth type or T stage. More recently, a technique of modified extended liver resection which aimed to reduce the removal of the amount of functional liver parenchyma has been described with a R0 resection rate of 92 % and an overall mortality rate of 7 % [81]. In this strategy, segment 4a was preserved in extended right hepatectomy and a modified extended left hepatectomy was performed by preserving the bile duct of segment 8 with its associated parenchyma on the cranial side. The authors also employed mesohepatectomy as an alternative to extended right hepatectomy for Bismuth type IIIa and IV tumors, when tumor infiltration into the ducts of segments 6 and 7 was limited and the right hepatic artery was not involved [81]. Third, even though minor central liver resection is technically more difficult than the other types of major liver resections because of the many intrahepatic ductal openings that need to be anastomosed, we have described a special technique of hepaticojejunostomy to solve this problem [62].

The lack of consensus on the extent of liver resection seems to arise mainly from the difficulty in precisely determining the extent of the proximal tumor preoperatively and intraoperatively. Kawasaki et al. argued against minor central liver resection and claimed that major hepatectomy should be performed for all patients with hilar cholangiocarcinoma because of the limitations of the currently available preoperative diagnostic modalities [60]. Some surgeons thought minor central liver resection should be limited strictly to patients with T2 tumor which has not invaded beyond any of the segmental hepatic ducts [44, 50, 62, 80]. Central minor liver resection carried out departing from these principles may result in poor survival outcomes. Indeed, minor central liver resections with curative intent should only be applied after precise anatomical assessment of the biliary tract with adequate assessment of the extent of tumor [44, 81]. The dilemma between major liver resection with potential postoperative liver failure and central minor liver resection with potential positive resection margins might be solved by advances in preoperative assessment. Concomitant use of three dimension and multiplanar reconstruction images using multidetector row computed tomography data can precisely detect both longitudinal and vertical tumor invasion [82]. This technique is noninvasive and can improve the curative resection rate, which might reduce the risk of positive margin even in minor liver resection. Sasaki et al. estimated length of proximal hepatic ducts using this technique, and found 17 in 18 hepatic ducts (94 %) were diagnosed negative [83]. However, this technique has not yet been evaluated in minor liver resections to our knowledge. Further advances in sensitivity of this technique are expected and may provide the hope to determine the extent of surgical resection for a tumor in a patient.

#### Left- or right-sided hepatectomy

The decision of whether right- or left-sided hemihepatectomy is indicated is made according to the predominant site of the lesion. In general, right hemihepatectomy can be applied to type IIIa tumors and IV tumors when the lesion is predominantly located in the right hepatic duct; whereas left hemihepatectomy can be applied to type IIIb tumors and IV tumors with left-sided predominance [60, 84, 85].

Right or extended right hemihepatectomy, the most radical surgical procedure, is routinely adopted by many surgeons on the basis of several anatomical considerations for patients with centrally situated hilar cholangiocarcinoma which can be treated by either combined right or left hemihepatectomy [8, 42, 51, 60, 86]. First, the extrahepatic part of the left hepatic duct is longer than the right hepatic duct, and the distance from the bifurcation to the sectional duct ramification is also much longer in the left liver. Second, the hepatic duct confluence lies on the right side of the hepatic hilum. Third, the right hepatic artery generally runs behind the common hepatic duct, and it is more likely to be invaded by tumor. Fourth, the left portal vein is also longer than the right portal vein. Finally, there are many anatomic variations which can jeopardize the safe performance of left-sided hepatectomy [87]. Therefore, right-sided hepatectomy is thought to be technically easier and has the additional advantage of radicality [42, 60, 88]. It is also emphasized that right-sided hepatectomy has superiority because it enabled en-bloc resection of the hepatic ductal confluence and its surrounding structures [89, 90]. In a right-sided hepatectomy predominated study, extended left hepatectomy was only occasionally performed as an alternative because of insufficient remnant liver volume [60]. Recently, Neuhaus et al. described the hilar en-bloc resection for hilar cholangiocarcinoma which comprises of resecting en bloc the portal vein bifurcation, the right hepatic artery, and liver segments 1 and 4 to 8, and showed its oncological superiority to the conventional combined major hepatectomy [91].

The major drawback of the right-sided hepatectomy is loss of a large volume of liver mass. Farges et al. reported a higher mortality rate after right-sided hepatectomy than left-sided hepatectomy [76]. Some surgeons also prefer left-sided hepatectomy because segment 4, an anatomical part of the left liver [81], is potentially invaded by tumor. By routinely resection segment 4, left-sided hepatectomy preserves more liver parenchyma than the right-sided approach (with only segment 2 and 3 remaining) [46].

Even though right-sided hepatectomy is the preferred approach for hilar cholangiocarcinoma [21], if there is a choice, in some series, left- or right-sided hepatectomies were carried out in a comparable number of patients [38, 52, 92]. In a recently large series published by Nagino et al. on 574 patients, left-sided hepatectomy contributed more than right-sided hepatectomy (51.8 vs. 38.3 %) [79]. In that cohort, type IV tumor took 45.5 % of all the cases and type III took 39.2 %. It seems that the author may prefer left-sided hepatectomy even for type IV tumors.

Shimuzu et al. performed left-sided hepatectomy for Bismuth type IIIb tumors in 88 patients and right-sided hepatectomy for type IIIa and IV tumors in 84 patients and showed equivalent operative curability and postoperative long-term survival between patients undergoing left-sided hepatectomy and right-sided hepatectomy [88]. Similarly, in a recent series reported by Lim et al., survival and recurrence rates after left hepatectomy were not significantly different from right hepatectomy in patients with type I and II hilar cholangiocarcinoma [61]. Further studies are required to identify the treatment strategy for type IV hilar cholangiocarcinoma between right- and left-sided hepatectomy.

### Combined vascular resection

The anatomical location of hilar cholangiocarcinoma is close to the portal vein bifurcation and the hepatic artery. These vascular structures are often invaded by tumor. The involvement of these vascular structures calls for combined vascular resection to achieve R0 resection. The indications for combined vascular resection include intraoperative suspicion of gross tumor invasion to the vessels [43, 93, 94], tight adherence of the tumor to the vessels during vascular skeletonization [8, 22, 55] and routine resection of portal vein in systematic radical surgery as advocated by some authors [10]..

**Fig. 4:**
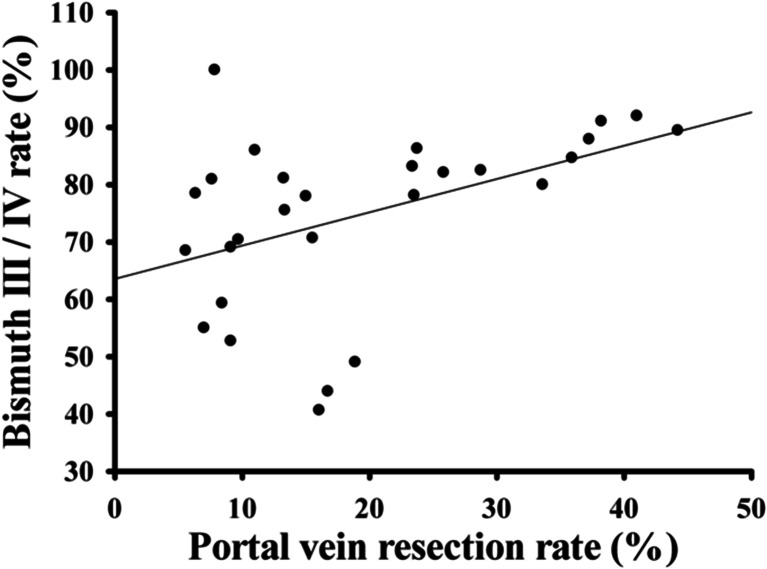
Portal vein resection rate significantly correlate with Bismuth type. Data were extracted from Table 1

Concomitant hepatic arterial resection and reconstruction should be performed with caution because it may result in higher morbidity and mortality rates but without any proven survival benefit [95]. Recent three meta-analyses draw similar conclusions that portal vein resection does not affect on postoperative mortality [95–97]. This consensus needs to be carefully interpreted. First, Wu et al. conducted the conclusion from subgroup analysis including studies from experienced surgeons and those published after 2007 [97]. Second, Abbas et al. draw the conclusion in an indirect manner. They found that the increase in mortality in patients who received vascular resection resulted from concomitant hepatic arterial resection, thus supposed that portal vein resection had no impact on postoperative mortality [95]. Another similar conclusion from these studies is that portal vein resection does not increase morbidity [95–97], and there is only occasional postoperative portal vein thrombosis (Table 3). Thus, portal vein resection can be performed safely. However, patients in portal vein resection cohort have lower 5-year survival rates. At first sight, the value of portal vein resection is limit for hilar CC patients with portal vein involvement. One should notice that patients who received portal vein resection had significantly higher rates of advanced disease (T3 and T4) when compared with patients without portal vein involvement [10, 95, 97], and they tended to have Bismuth III or IV tumors (Fig. 4). The importance of portal vein resection should be revealed by investigating the impact of portal vein resection on the surgical results for patients with the same tumor stage and Bismuth type.

**Table 3:** Complications related to portal vein resection

Authors	Published year	Resections (*n*)	PVR (*n*)	Outcomes of PVR
Klempnauer et al.	1997	151	39	Three portal vein thrombosis
Neuhaus et al.	1999	80	23	Do not associate with mortality
Gerhards et al.	2000	112	10	Significant predictors of increased mortality.
Capussotti et al.	2002	36	5	Do not associate with morbidity and mortality
Kawasaki et al.	2003	79	5	Do not associate with survival
Seyama et al.	2003	58	9	Do not associate with survival
Dinant et al.	2006	99	7	Do not associate with morbidity and mortality
Hasegawa et al.	2007	49	3	Do not associate with survival
Konstadoulakis et al.	2008	59	14	Do not associate with morbidity and mortality
Yong et al.	2009	51	21	Zero mortality, and 1 portal vein thrombosis
Lee et al.	2010	302	40	Zero mortality, and 1 portal vein thrombosis
Igami et al.	2010	298	111	Five portal vein thrombosis
Hemming et al.	2011	95	42	Do not associated with mortality
Nagino et al.	2012	574	206	Do not associated with mortality
de Jong et al.	2012	305	51	Increase perioperative risk (mortality)
Song et al.	2013	230	22	One portal vein thrombosis
Gomez et al.	2014	57	5	One portal vein thrombosis
Yu et al.	2014	119	25	Had no effect on patient survival
Tamoto et al.	2014	49	36	Do not associate with post-operative complications.

*PVR* portal vein resection

The histological involved margin status seems more important than the presence of direct invasion/involvement of portal vein for long-term overall survival [10]. Even the role of portal vein resection on R0 margin rates is still controversial [95–97], logically portal vein resection allows patients who had advanced tumor a chance to achieve a better histological result. Therefore, when portal vein invasion is suspected, combined portal vein resection should be carried out. This conclusion is further supported by a recent international, multicenter, retrospective study on a large cohort of patients [10].

The reported rates of actual tumor invasion into the resected vessels varied from 22 to 100 % [8, 10, 90, 93, 94, 98–100]. This indicate that it is difficult to determine the actual status of vascular involvement preoperatively or intraoperatively and that the indication of portal vein resection is quite variety. Therefore, some Japanese surgeons advocated routine portal vein resection for hilar cholangiocarcinoma to achieve better long-term survival [4, 8, 91, 101]. However, the benefit of routine portal vein resection requires further evidence to support [5, 13, 95].

## Conclusion

Treatment for hilar cholangiocarcinoma remains challenging. In order to improve surgical outcomes for patients with hilar cholangiocarcinoma, strategy of surgical resection with curative intent has improved over the years. Some consensuses have been reached, including R0 margin achievement, routine caudate lobe resection, combination of partial hepatectomy, and portal vein resection when involved by tumor. However, there are still several controversial issues need to be further clarified. Improvement of hepatobiliary imageological technique that can provide more accurate information of the tumor extent preoperatively will offer help. Due to the rarity of hilar cholangiocarcinoma, prospective randomized studies can only be carried out in multiple centers.
